# First record of *Triatoma longipennis,* Usinger, 1939 (Hemiptera: Reduviidae: Triatominae) in Tecozautla, Hidalgo

**DOI:** 10.1590/0037-8682-0078-2023

**Published:** 2023-07-24

**Authors:** Nancy Rivas, Alberto Antonio-Campos, Julio Noguez-García, Ricardo Alejandre-Aguilar

**Affiliations:** 1 Instituto Politécnico Nacional, Escuela Nacional de Ciencias Biológicas, Ciudad de México, México. Instituto Politécnico Nacional Escuela Nacional de Ciencias Biológicas Ciudad de México México; 2 Servicios de Salud Hidalgo, Área de Entomología, Laboratorio Estatal de Salud Pública de Hidalgo, Pachuca de Soto, Hidalgo, México. Servicios de Salud Hidalgo Área de Entomología Laboratorio Estatal de Salud Pública de Hidalgo Pachuca de Soto Hidalgo México

**Keywords:** Triatoma longipennis, Chagas disease vector, Trypanosomiasis, Distribution

## Abstract

**Background:**

We report the presence of *Triatoma longipennis* for the first time in two localities in Hidalgo, Mexico.

**Methods:**

This study was conducted at Tecozautla municipality, Hidalgo. Collection was performed in April 2022.

**Results:**

We collected eight triatomines from Guadalupe: two fourth-instar nymphs, three fifth-instar nymphs, one female, and two males. In San Miguel Caltepantla, a female was collected inside a dwelling. One sample tested positive for *Trypanosoma cruzi*.

**Conclusions:**

These findings suggest the need to investigate the dynamics of this species with respect to the inhabitants of the study area.

Chagas disease is an important but neglected tropical disease caused by the hemoflagellate parasite *Trypanosoma cruzi* (Chagas, 1909) (Kinetoplastida, Trypanosomatidae) and is transmitted via hematophagous insect vectors. The World Health Organization has estimated that as many as 8 million people in Mexico, Central America, and South America have Chagas disease. Although different forms of transmission exist in endemic countries, the main route of transmission is through the feces and urine of triatomines^1^.

There are currently 156 known living triatomine species and 3 fossil species, which are grouped into 18 genera and 5 tribes^2^^,^^3^. In Mexico, 35 triatomine species have been reported and are grouped into 8 genera^3^^,^^4^^,^^5^. The *Triatoma* genus, consisting of 24 species (six formerly called *Meccus*), is the most abundant and widely distributed genus.

Hidalgo is considered an endemic state for Chagas disease, and the presence of *Triatoma barberi* Usinger, 1939, *T. dimidiata* (Latreille, 1811), *T. mexicana* (Herrich-Schaeffeer, 1848), and *T. gestaeckeri* (Stål, 1859) has previously been described^6^. 

*Triatoma longipennis,* Usinger, 1939 (formerly called *Meccus longipennis*) is considered the main species responsible for the transmission of *T. cruzi* in northern, western, and central Mexico^7^. Its current geographical distribution includes 10 of 32 Mexican states: Aguascalientes, Chihuahua, Colima, Durango, Guanajuato, Jalisco, Michoacan, Nayarit, Sinaloa, and Zacatecas^8^. This triatomine species is considered peridomiciliary, with percentages of natural infections ranging between 21.7% and 33.3% in different regions^9^. In addition, *T. longipennis* belongs to *Phyllosoma* complex, formed by *T. bassolsae* Alejandre-Aguilar et al.,1999; *T. mazzottii* Usinger, 1939, *T. mexicana*; *T. pallidipennis* (Stål, 1872), *T. phyllosoma*, and *T. picturata* Usinger, 1939, among which the possibility of natural crossbreeding has already been reported^10^. 

This study reports, for the first time, the presence of *T. longipennis* naturally infected with *Trypanosoma cruzi* in two localities of Tecozautla, a municipality in Hidalgo, Mexico.

This study was conducted in the Tecozautla municipality, which is one of the eighty-four municipalities in Hidalgo, Mexico, and is located in the Neovolcanic Axis region within the sierras and plains subregions of Querétaro and Hidalgo. The predominant climate in Tecozautla is semi-warm, and temperate semi-dry with an average annual temperature of 17 °C and a total annual rainfall of 517 mm. Guadalupe (1968 masl) and San Miguel Caltepantla (1849 masl) are divided by a mountainous formation that separates them by a straight-line distance of 8.17 km (Figure 1).

**FIGURE 1:**
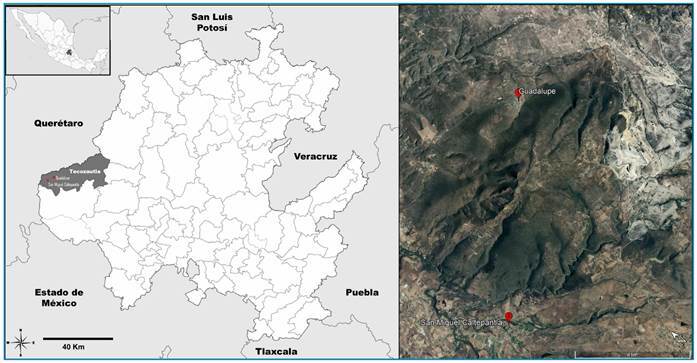
Distribution of *Triatoma longipennis* in Guadalupe and San Miguel Caltepantla, Tecozautla, Hidalgo State. The red spots indicate the location of the sampling sites.

Triatomines were collected manually (12/04/2022 to 28/04/2022) according to Mexican norm (NOM-032-SSA2-2014). Each sample placed in a plastic container that was labeled for further experiments. 

Triatome identification was performed at the Laboratory of Medical Entomology of “Escuela Nacional de Ciencias Biologicas, Instituto Politecnico Nacional,” Mexico City, according to the keys of Lent and Wygodzinsky^11^. To identify triatomines infected with *T. cruzi*, feces from each triatomine were obtained by spontaneous defecation after feeding laboratory-reared New Zealand white rabbits. Fecal samples were mixed with 1× phosphate buffered saline (pH 7.2) and examined at 400× using an optical microscope (LEICA DM500) for the presence of *T. cruzi*. Smears with Giemsa staining were performed to verify the characteristic morphology of *T. cruzi* and confirmed by PCR using the protocol reported by Padilla-Valdez *et al*.^12^ Parasites were isolated in LIT (Liver Infusion Tryptose) medium supplemented with antibiotics and antifungals (data not shown). All the triatomines were maintained in the insectary at 28 °C with 60% relative humidity.

All field-collected triatomines were identified as *T. longipennis* (Figure 2). The collected female and male *T. longipennis* had a length of 40 and 38 mm; a pronotum width of 8 and 7 mm; and an abdomen width of 18 and 15 mm, respectively. Females had wide bodies. Overall, the body was dark brown or black in color, with orange spots on the neck and abundant 0.1 mm long-setae. The head was black and weakly granulose with numerous forwardly inclined setae. The head was two to two-and-one-half times as long as it was wide (1:0.4) and varied from longer to very slightly shorter than the pronotum (1:0.85) and black rostrum. The pronotum and scutellum was uniformly black and dark, respectively. The corium was black, with large triangular, orange-colored basal markings and a small subapical orange spot. The connexivum was black, with each segment in the posterior half or third exhibiting irregularly shaped orange markings adjoining the abdominal margins (Figure 2).

**FIGURE 2:**
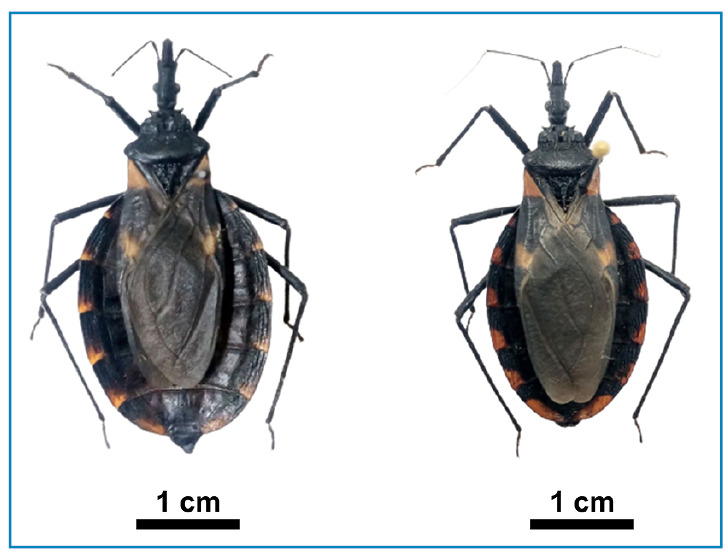
Female and male samples of T*riatoma longipennis* from Guadalupe and San Miguel Caltepantla, Tecozautla, Hidalgo State. Scale bar = 1 cm.

In Guadalupe (20°31’45.79’’ N; -99°33’39.53’’ W), one of the *T. longipennis* specimens (male) was collected inside a dwelling under a bed. Microscopic analysis revealed the presence of *T. cruzi* in the feces of the triatomine. Other triatomines (one male, one female, three fifth-stage nymphs, and two fourth-stage nymphs) from Guadalupe were collected from the peridomicile inside an animal stall. One of the fifth-stage nymphs collected in this locality molted into the adult stage two days after arriving at the laboratory. The only female triatomine collected inside the household (under the bed) in San Miguel Caltepantla (20° 28’ 56.96’’ N; -99° 37’ 32.32’’ W) tested negative for *T. cruzi* infection.

These results demonstrate the presence of *T. longipennis* infected with *T. cruzi* in regions where the species had not been previously reported, in addition to determining its presence in peri- and intra-domestic environments. Hidalgo State does not border any of the states where *T. longipennis* has been previously reported. This finding suggests several possible scenarios, one being that this species was passively introduced, such as in the recently reported case of *Triatoma infestans* in Mexico^5^, and another being that this species is present in at least one neighboring state. The latter scenario is most likely related to the species’ presence in Queretaro State, which borders Hidalgo to the south and Guanajuato to the north, and where *T. longipennis* and *T. mexicana* have already been found^13^. Nevertheless, future studies are necessary to expand the search area and conduct a phylogeographic analysis that will allow the dynamics of this triatomine species to be elucidated with respect to different regions where its presence has been reported.

In addition, combining the findings of this study with those of a previous report^14^, we determined that *T. longipennis* and *T. pallidipennis* are sympatric in the Guadalupe locality. These data are important because both species of triatomine belong to the *Triatoma phyllosoma* complex and the existence of natural hybrids between both species has been demonstrated^10^; however, their epidemiological traits have not been fully elucidated.

The presence of *T. barberi*, *T. dimidiata*, *T. mexicana*, and *T. gerstaeckeri* has been commonly described in Hidalgo. However, recent studies have also provided morphological and phylogenetic evidence of the presence of the cryptic species *T. mexicana*^13^ and *T. pallidipennis*^15^. Thus, with the added presence of *T. longipennis*, there are currently eight known species of triatomines in Hidalgo, with several being of public health importance, such as *T. barberi*, *T. dimidiata*, *T. mexicana*^6^, *T. longipennis*^7^, and *T. pallidipennis*^15^.

This study showed that although the presence of *T. longipennis* has not been previously reported in some geographic regions, this does not imply that it is absent. However, more intensive studies are necessary to ascertain the current distribution of triatomine species in Mexico. Furthermore, it is necessary to accurately identify and provide evidence to design and improve sound vector control programs based on the behavior of each triatomine species. 

## References

[1] 1. World Health Organization (WHO). Chagas disease (American trypanosomiasis). 2022. [update 2022 November 28; cited 2022 December 2]. Available from: Available from: https://www.who.int/news-room/fact-sheets/detail/chagas-disease-(american-trypanosomiasis) .

[2] 2. Gil-Santana HR, Chavez T, Pita S, Panzera F, Galvão C. *Panstrongylus noireaui*, a remarkable new species of Triatominae (Hemiptera, Reduviidae) from Bolivia. Zookeys. 2022;1104:203-25. 10.3897/zookeys.1104.81879 PMC9848746 36761929

[3] 3. Téllez-Rendón J, Esteban L, Rengifo-Correa L, Díaz-Albiter H, Huerta H, Dale C. *Triatoma yelapensis* sp. nov. (Hemiptera: Reduviidae) from Mexico, with a Key of *Triatoma* Species Recorded in Mexico. Insects. 2023;14(4):331. 10.3390/insects14040331 PMC10142269 37103146

[4] 4. Ramsey JM, Peterson AT, Carmona-Castro O, Moon-Llanes D, Nakazawa Y, Butrick M, et al. Atlas of Mexican Triatominae (Reduviidae: Hemiptera) and vector transmission of Chagas disease. Mem Inst Oswaldo Cruz. 2015;110(3):339-52. 10.1590/0074-02760140404 PMC4489471 25993505

[5] 5. Martínez-Hernández F, Villalobos G, Montañez-Valdez OD, Martínez-Ibarra JA. A New Record of the Introduced Species *Triatoma infestans* (Hemiptera: Reduviidae) in Mexico. J Med Entomol. 2022;59(6):2150-7. 10.1093/jme/tjac078 35716079

[6] 6. Antonio-Campos A, Cuatepotzo-Jiménez V, Noguéz-García J, Alejandre-Aguilar R, Rivas N. Distribution of triatomine (Hemiptera: Reduviidae) vectors of Chagas disease in the state of Hidalgo, Mexico. J Vector Ecol. 2019;44(1):179-86. 10.1111/jvec.12342 31124229

[7] 7. Brenière SF, Waleckx E, Magallón-Gastélum E, Bosseno MF, Hardy X, Ndo C, Lozano-Kasten F, et al. Population genetic structure of *Meccus longipennis* (Hemiptera, Reduviidae, Triatominae), vector of Chagas disease in West Mexico. Infect Genet Evol. 2012;12(2) 254-62. 10.1016/j.meegid.2011.11.003 22142488

[8] 8. Martínez-Hernandez F, Villalobos G, Martínez-Ibarra JA. Population structure and genetic diversity of *Triatoma longipennis* (Usinger, 1939) (Heteroptera: Reduviidae: Triatominae) in Mexico. Infect Genet Evol . 2021;89:104718. 10.1016/j.meegid.2021.104718 33444857

[9] 9. Espinoza-Gómez F, Maldonado-Rodríguez A, Coll-Cárdenas R, Hernández-Suárez CM, Fernández-Salas I. Presence of Triatominae (Hemiptera, Reduviidae) and Risk of Transmission of Chagas Disease in Colima, México. Mem Inst Oswaldo Cruz . 2002;97(1):25-30. 10.1590/s0074-02762002000100002 11992142

[10] 10. Martínez-Hernandez F, Martínez-Ibarra JA, Catalá S, Villalobos G, Torre P de la, Laclette JP, et al. Natural Crossbreeding between Sympatric Species of the *Phyllosoma* Complex (Insecta: Hemiptera: Reduviidae) Indicate the Existence of Only One Species with Morphologic and Genetic Variations. Am J Trop Med Hyg. 2010;82(1):74-82. 10.4269/ajtmh.2010.09-0272 PMC2803513 20064999

[11] 11. Lent H, Wygodzinsky P. Revision of Triatominae (Hemiptera, Reduviidae) and their significance as vectors of Chagas Disease. Bull Amer Mus Nat Hist. 1979;163(3):123-520.

[12] 12. Padilla-Valdez JM, Antonio-Campos A, Arias-del-Angel J A, Rivas N, Alejandre-Aguilar R. Susceptibility dynamics between five Trypanosoma cruzi strains and three triatomine (Hemiptera: Reduviidae) species. J Vector Ecol . 2021;46(1):82-95. 10.52707/1081-1710-46.1.82 35229585

[13] 13. Rivas N, Martínez-Hernández F, Antonio-Campos A, Sánchez-Cordero V, Alejandre-Aguilar R. Genetic diversity in peridomiciliary populations of *Triatoma mexicana* (Hemiptera: Reduviidae: Triatominae) in central Mexico. Parasitol Res. 2022;121(10):2875-86. 10.1007/s00436-022-07608-2 35930043

[14] 14. Alejandre-Aguilar R, Antonio-Campos A, Noguéz-García J, Rivas N. *Triatoma pallidipennis* (Stål, 1872) (Hemiptera: Reduviidae) and its potential for infestation in Tecozautla, Hidalgo state, Mexico. J Vector Ecol . 2023;48(1):1-6. 10.52707/1081-1710-48.1.1 37255353

[15] 15. Cruz DD, Arellano E. Molecular data confirm *Triatoma pallidipennis* Stål, 1872 (Hemiptera: Reduviidae: Triatominae) as a novel cryptic species complex. Acta Trop. 2022;229:106382. 10.1016/j.actatropica.2022.106382 35189124

